# ‘Men value their dignity’: securing respect and identity construction in urban informal settlements in South Africa

**DOI:** 10.3402/gha.v7.23676

**Published:** 2014-04-08

**Authors:** Andrew Gibbs, Yandisa Sikweyiya, Rachel Jewkes

**Affiliations:** 1 Health Economics and HIV/AIDS Research Division (HEARD), University of KwaZulu-Natal, Durban, South Africa; 2 Gender and Health Research Unit, Medical Research Council, Pretoria, South Africa

**Keywords:** masculinity, HIV, violence, gender, multiple sexual partners, livelihoods, IPV, unemployment

## Abstract

**Background:**

Urban informal settlements remain sites of high HIV incidence and prevalence, as well as violence. Increasing attention is paid on how configurations of young men's masculinities shape these practices through exploring how men build respect and identity. In this paper, we explore how young Black South Africans in two urban informal settlements construct respect and a masculine identity.

**Methods:**

Data are drawn from three focus groups and 19 in-depth interviews.

**Results:**

We suggest that while young men aspire to a ‘traditional’ masculinity, prioritising economic power and control over the household, we suggest that a youth masculinity emerges which, in lieu of alternative ways to display power, prioritises violence and control over men's sexual partners, men seeking multiple sexual partners and men's violence to other men. This functions as a way of demonstrating masculinity and their position within a public gender order.

**Discussion:**

We suggest there are three implications of the findings for working with men on violence and HIV-risk reduction. First, there exist a number of contradictions in men's discourses about masculinity that may provide spaces and opportunities for change. Second, it is important to work on multiple issues at once given the way violence, alcohol use, and sexual risk are interlinked in youth masculinity. Finally, engaging with men's exclusion from the capitalist system may provide an important way to reduce violence.

Rapid urbanisation has led to burgeoning informal settlements, as cities and states have been unable to effectively create permanent infrastructure for growing populations (1, 2). WHO and UN-HABITAT estimated in 2010 that 63% of urban dwellers in sub-Saharan Africa lived in informal settlements (3). In South Africa, the 1980s saw a rapid growth of urban informal settlements, when the apartheid government stopped controlling the mobility of the Black majority with repeal of the influx control legislation, but failed to meet permanent housing needs (4). Current estimates for South Africa suggest that 4.4 million people live in informal settlements, approximately 23% of all households (5). In eThekwini district, KwaZulu-Natal – the location of this study and the third largest city in South Africa – an estimated 25% of the population live in informal housing (5).

Urban informal settlements are often settings with high levels of violence, poverty, poor health, and HIV (4, 6–8). UNAIDS estimates that 28% of people living with HIV/AIDS in southern and eastern Africa live in 14 cities in the region (approximately 15% of the global epidemic) and in South Africa the HIV prevalence in informal settlements is twice that of people in formal housing (9, 10). While there remains little comparable data on rates of gender-based violence in urban and rural areas (11), one study from Cape Town, South Africa, explored rates of homicide within the city, comparing different settlement ‘types’ and showed informal urban settlements had rates over four times that of wealthier, formal settlements (3).

There has been considerable debate about why urban informal settlements have particularly high levels of violence and ill-health and the role of place in health outcomes more generally (12). One strand of this argument emphasises the experience of living in high-density communities, leading to stress and an inability to control aspects of life, as a key factor shaping violence (11). Another argument is that in informal settlements there is less social cohesion, caused by poverty and mobility, creating less stable forms of power, in which violence becomes a necessary resource to wield as previously stable configurations of power – particularly gender power – get challenged (3, 11).

## Masculinities and violence

Globally, researchers are increasingly studying constructions of masculinity, including men's perpetration of violence, which place them and their partners at increased risk of acquiring HIV (13, 14). Firmly located within a critique of gender inequalities, studies have observed and sought to explain the clustering of men's violence and HIV-risk practices (15). In South Africa, a representative population-based study of South African men found those who had been violent to a partner to have less gender equitable masculinities, more likely to have raped and more likely to have engaged in transactional sex (16). Specifically in the study, among those under 25 years, those who had been violent to a partner had a higher prevalence of HIV (16). Similar links between violence, rape, and gender inequitable masculinities have also been shown in the Asia–Pacific region (17) and Latin America (18, 19).

Theorising this clustering of risk, violence and gender inequitable masculinities researchers have largely drawn on Connell's (20) notion of hegemonic masculinity, building a relational construction of gender inequalities (21). Within a context of patriarchal privilege, Connell argues that in any social setting there is a collectively held understanding of ideal male practices. The majority of men view the ideal as an aspiration, something that influences their practices and structures men's understandings of themselves and their behaviours, without necessarily being achievable or desired in its entirety for all men (20). In response, men construct a range of masculinities allowing them to establish viable alternatives to the hegemonic masculinity, while at the same time often supporting its overall logic (20).These hegemonic ideals also influence the behaviour of women as although they are subordinated by men, they shape their views of a desirable ideal and thus men who do not aspire to adopt the hegemonic masculinity may be penalised in their attractiveness to women.

Men's behaviours, including violence and HIV-risk-related practices, can be understood as men attempting to position themselves both individually and publically in relation to hegemonic masculinity, which forms a gender hierarchy (19, 22, 23). Critical men's studies also point towards how such health behaviours actively constitute forms of masculinity (24). From a social–psychological perspective, some researchers are concerned with how these broad macro-processes become embedded in individual's psyches and how these are internalised and resisted (25). In the context of high levels of poverty, a strong argument has been made that young men construct a subordinated masculinity, focused on heterosexual performance and violence as a way of building their sense of self-worth and positioning themselves within the gender and broader social order of these socially subordinated spaces (20, 26, 27). Less often commented on is how gender hierarchies intersect with age hierarchies and violence can often be seen as situated at the intersection of these axes as well (27).

While much work accentuates men's power, dominance, and use of violence against women and other men, another set of work emphasises the emotional lives and vulnerability many of these men living in poverty feel (28, 29). This has led some to suggest that men's violence emerges from a profound sense of powerlessness (30) with men seeking power in ways that are accessible to them and socially condoned. Some researchers have sought to trace men's ‘long histories of violence’, through exploring men's childhoods that are harsh and leading to ‘attachment disorders’, which tend to reduce men's empathy and guilt. In so doing, they suggest that the patterns of violence and other risk behaviours are setup in childhood psychological development processes but then enabled through social process and contexts – such as patriarchy – to support men's violence against women and other men (31).

## Masculinities in South Africa

Within South Africa a number of ethnographies have sought to understand how men construct and sustain masculine identities and respect in a variety of contexts. Hunter's (4) work suggests that in the 1970s and 1980s a new ‘traditional masculinity’ emerged for Black South African men employed in working class jobs as industrialisation occurred. This masculinity centred on a benign heterosexual patriarchy in which masculine respect was underpinned by male economic provision (4). This reworked older notions of masculinity locating them in urban settings. Central to this was men's ability to provide for a household with homes becoming a measure of masculinity (4, 32). Male power was also articulated through asserting social control over women and children. According to Hunter (4) this masculinity continues to dominate the gender hierarchy for many working class Black South Africans, potentially forming a hegemonic masculinity (20).

As much research on masculinities has emphasised, for the majority of men (if not all), the ‘hegemonic masculinity’ cannot be achieved and a multiplicity of masculinities flourish (21). Studies in South Africa have explored alternative ways of building masculine identity and respect. Reihling (33) looks at how men living with HIV construct new forms of what he calls ‘relational dignity’ through health activism, creating a new form of masculinity in so doing. A small number of studies have sought to explore youth masculinities and health in contemporary South Africa. Wood and Jewkes (34), for instance, in the Eastern Cape Province of South Africa argue that economic marginalisation of young men has led to a distinctive youth masculinity emerging, where masculinity became centred on controlling main female sexual partners, with violence used if necessary. Similarly, Ragnarsson et al.'s (8) work in peri-urban communities emphasises how small male groups are the central locus for this production of a patriarchal youth masculinity, in which men in lieu of alternative sources of power and dignity turn to seeking multiple sexual partnerships as a way of securing their masculinity among other men. While Hunter's work (4, 35) also exploring younger men, emphasises how young men negotiate the tensions between their expected roles as providers in romantic relationships and their lack of economic power through subtle negotiations and an emphasised heterosexuality.

In this study, we build on this body of work to explore how young Black South African men, living in contexts of poverty in urban informal settlements, seek to construct, and sustain a viable sense of respect and masculine identity through their relationships with others focused on the intersections of sexuality and violence. We are concerned throughout with how men evaluate themselves and position themselves within gender and age hierarchies.

## Methods

### Setting

The young men in the study lived in two urban informal settlements in eThekwini District, KwaZulu-Natal, South Africa. Broadly informal settlements in South Africa have poor services; 2001 data suggested that only 26% of dwellings in informal settlements had piped water in their dwelling or yard and 32% had electricity (5) and the two settlements reflected this. The majority of young men came from a slightly older and more established settlement, Little Japan. Little Japan had a number of government provided single room houses (called RDP-houses) sitting alongside smaller shacks and single room dwellings. It was located alongside a main highway, which ran past a shopping mall and large township, approximately 10 min away by taxi. There was a regular public taxi to the centre of Durban taking about 25 min. Despite this Little Japan's roads were primarily untarred and there was little formal electricity and no inside toilets. The second community was Mbazwana and significantly poorer than Little Japan. This was a new settlement, only settled in the previous 10 years, located on a steep hillside. Transport links into Durban and to industrial areas were weak. Residents of Mbazwana had to catch two public taxis to central Durban, taking about 45 min. There was also no formal electricity, pathways, or toilets in Mbazwana.

### Participants

Men were aged between 18 and 27 years, with the majority under 25. A few had formally finished education with a high school qualification, but most had exited education early, and few had further skills training. None of the men in the study had permanent work; rather the majority relied on temporary formal work (primarily shop work or construction), informal work (such as selling small items at the side of the road or working on public taxis), or a variety of illegal activities (selling drugs or petty crime). This work was poorly paid and highly precarious. Nationally representative household data from 2006 highlight the casualised nature of work in informal settlements (36). These data also suggest that average wages in informal settlements were R1,703 per month compared to R2,945 in formal housing (36). Many of the men also relied on their family to support them financially. All of the men reported that they had a main female partner at the time of the interviews and a number had a child with this partner or a previous partner.

### Data collection

Data for this paper come from three focus group discussions (FGDs) conducted with 44 men and 19 in-depth interviews (IDIs) conducted over 2 months in 2012. FGDs enable collectively held views and understandings of salient issues to emerge – what we may call public transcripts – while IDIs enable the complexities and ambivalences of real lives to emerge, without men feeling compelled to construct public identities (37).

Data were collected at baseline for a formative evaluation of a behavioural and structural intervention – Stepping Stones and Creating Futures (38). Participants were recruited by Project Empower, an NGO based in eThekweni, which ran the intervention. Open community meetings were held at which the intervention was explained and flyers circulated. As such, participants self-selected to participate in the study. A convenience sample was used for the FGDs; we approached all of the men who enrolled in the study in the first three days and requested their participation in FGDs, 44 men agreed. While FGDs were large (ranging from 12 to 20 participants), it enabled an exchange of views and ideas to emerge. As Tang and Davis (39) suggest there is no optimal size for FGDs as long as sufficient time and facilitation is in place to enable a meaningful exchange of ideas to occur. From the 110 men who enrolled in the intervention, we randomly selected 20 men to participate in IDIs – 19 men agreed. We randomly chose men for IDIs as we then followed men up over the course of 1 year to understand their overall experience of the intervention and did not wish to introduce bias into our selection.

The IDIs and FGDs covered similar topics. They focused on the intersection between masculinities and livelihoods and how this shaped men's lives and relationships. Specifically they included discussions on how men made money and survived on a daily basis and what they aspired to do in the future. Questions probed what men felt it meant to be a man in their community and whether they achieved this or not. The topic guide then moved onto relationships with women, especially sexual partners before asking about violence in the community and in their relationships. IDIs typically lasted about 45 min, ranging from 20 min to 1.5 hours. FGDs lasted between 1 and 1.5 hours. All FGDs and IDIs were conducted in isiZulu, the dominant language in the study locations, and were digitally recorded and translated and transcribed by the male fieldworker who undertook them.

### Data analysis

Thematic content analysis was conducted drawing on Attride-Stirling's approach of thematic network analysis (40). Broadly, transcripts were read repeatedly before initial codes were developed (based on words or short ideas) (41). Codes were then clustered into groups focused on how men understood respect and sought to achieve it. Triangulation was achieved by comparing and contrasting FGDs and IDIs to examine both public and private understandings and expressions of masculinity. These were then centred on two networks identified as ‘traditional masculinity’ and ‘youth masculinity’. Such an approach allows the researcher to make connections between different ideas and link to theory rather than simply describe data (40).

### Ethics

Ethical approval was given by the South African Medical Research Council (EC003-2/2012) and the University of KwaZulu-Natal's Human and Social Science Ethics Committees (HSS/0789/011 and HSS/1273/011D). Written informed consent was obtained from all participants. The names of study participants and locations have been replaced by pseudonyms to protect the identity of the participants. No payment was given to participants for participating in the intervention or FGDs. However, for IDIs a small meal was bought by the research assistant and shared as a way of building rapport.

## Findings

The men identified with a ‘traditional’ masculinity premised on economically providing in relationships, in which men were positioned as benevolent patriarchs. Yet young men's inability to secure work, left them socially positioned as children. As a reaction to this, the men were drawn to a particular youth masculinity that emphasised respect through violence against partners, control of partners, seeking multiple sexual partners, and violence against other men.

### ‘Traditional’ masculinity

Young men aspired to a ‘traditional’ masculinity, closely linking masculinity to provision for a family and partner and control over them. Gwedi, for instance, saw manliness as embodied by having a home and control over the family:

Interviewer: What characteristics does a person need to have in order to be described as a man in your community?

Gwedi: You know there is no other way my brother, you must have a wife, a house, and money, and again to see how well behaved you are when you are a man you must be straight [strict]. (IDI, 24, petty drug seller)^1^

Economic independence was prized by men as it enabled them to set up a household. Borrowing money, rather than working for it, as many of the young men did, was seen as a sign of failure as Thokozani commented:

Interviewer: What does it mean to be a man?

Thokozani: I have to be responsible and be independent, respectable in the community.

Interviewer: What do you mean by independence?

Thokozani: Like having my own house. Not being a person that is always borrowing money. (IDI, 19, supported by parents)

Among informants, the use of violence to settle disputes among men was discussed. For some violence, owning guns and knives and a willingness to wield violence remained important. However, for most the ‘traditional’ masculinity was gentler and prioritised aspects of love, kindness, and engagement with children, as well as limiting violence as Bongani emphasised:

Interviewer: What makes a successful man in your community?

Bongani: It is the way he carries himself [the way he behaves], having respect…

Interviewer: How is he to his family?

Bongani: He is a disciplined man. He has a wife and it does not mean just because you have a wife you cannot wash dishes, a man is able to talk well with his wife, not violently, and his kids love him as a father. (IDI, 25, informal shop)

Broadly, men in the study still aspired to a ‘traditional’ masculinity in which power was conferred to them through economic independence and social dominance, essentially creating a hegemonic masculinity.

### Men without respect

Young men, however, were aware that the ‘traditional’ masculinity was aspirational and something they struggled to achieve. Men described how they were often highly dependent on their families for financial support – primarily mothers or grandmothers. As Thabo described, this dependency undermined his sense of confidence and masculinity:

Thabo: The thing is my grandmother, she buys me food, she dresses me and she supports my child. Now to think of asking her for money, let's say me and my friends want to buy booze and party, to me that is a problem.

Interviewer: Has asking money from your granny caused you any problems?

Thabo: I'm too dependent on her, whilst I should be independent. (IDI, 23, piece work)

Without formal work, young men spent much time ‘hanging around on streets’. This enabled public ‘devaluation’ of the men by others in the community, who did not take them seriously as they did not work. As Mboniswa suggested, men without work were viewed as useless, as less than men, as they could not support a family:

Interviewer: How do they view a man who does not work?

Mboniswa: They view him as a useless man. Like someone you cannot depend on or look up to. They would ignore him, not take him seriously and look down upon him, or as someone that does not exist in the community. (IDI, 23, informal work)

Within the public gender and age hierarchy that existed within the communities, a lack of access to work placed young men low down. Indeed many, including Wiseman, stressed how they were seen as children as they did not conform to the ideals of masculinity:

Interviewer: How does the community treat you if you don't meet the characteristics of being that man?

Wiseman: Okay, yes, you are undermined. Like you are just a man because you wear pants [trousers] nothing more. You are looked down upon, even little boys undermine you, they treat you like you are at their age, because you are useless. (IDI, 18, temporary work)

Of particular concern for young men was their inability to provide in sexual relationships, as they felt was expected of them. Sandile described both the frustration and embarrassment that was caused when he could not provide basic items and how women looked down upon young men like himself:

Interviewer: What problems are there for a man when he does not have money?

Sandile: Most of the time women depend on men, so if you are a man and you don't have money, even when a woman is asking for something to wear or a perfume and you are not able to provide with that, it becomes a problem. It is an embarrassment.

Interviewer: What happens to you as a man when that happens?

Sandile: Your dignity is crushed and women badmouth you, like saying: ‘that man is just using me, he does not give me money, he doesn't do anything for me, he is just using me [for sex].’ (IDI, 24, temporary jobs)

Within urban informal settlements, young men were acutely aware of how others positioned them within the gender hierarchy and how they were positioned as children for failing to achieve what was expected of men.

### Building respect in informal settlements

In their communities, men struggled to establish themselves both as men in public settings and build their own sense of self-confidence and respect. In turn, men sought to construct an alternative identity predicated on the sources of power that they could access, primarily located around heterosexuality and violence. We identify four main aspects informing a dominant youth masculinity: 1) men's main sexual relationships, 2) violence and control over female partners, 3) having multiple partners and thus demonstrating desirability to women, 4) public violence. Each of these, in their own ways, enabled men to achieve a sense of respect in public and private contexts.

#### Men's main long-term sexual relationships

The majority of men said they had a long-term female sexual partner. As men spoke about these relationships, they sought to frame them in similar terms to how they had spoken about relationships within the ‘traditional’ masculinity they aspired towards, even if they could not achieve this. Almost all interviewees identified a woman they saw as a main partner, often someone they had a child with, and specifically someone they saw as having a future together with. They were able to distinguish these women and the relationships they had with them, from other relationships they had with other women, which were often shorter and more focused on sexual exchange.

Men were emotionally invested in these long-term relationships. Many reflected on how they would feel if these relationships ended, emphasising the emotional pain they would feel. Gwedi had two girlfriends; the first was his main partner with whom he had a child. The second was a younger woman who he saw occasionally. He described the different responses he would feel when asked to imagine what would happen if these relationships ended:

Interviewer: If one of your girlfriends wanted to leave you, what would happen? Let's say your baby mama [main partner and mother of his first child]?

Gwedi: Without a reason?

Interviewer: Whether or not without a reason. I want to know what would happen if one of them wanted to leave you?

Gwedi: I would be sad if it is my baby mama because you know I have invested my future with her since I want to go far with her. My heart would be broken but I would try and ask her not to leave me, but everything would be up to her because the person with the last decision would be her.

Interviewer: And the second one?

Gwedi: The second one if she wanted to leave me?

Interviewer: Yes.

Gwedi: The second one if she wanted to leave me it's not like I would be too heartbroken. Though I would be sad because she is the one close by for booty call [sex], I must say that would be sad in that sense, but she is not like that important to me. (IDI, 24, petty drug seller)

Men placed significant emphasis on trust and love in main relationships, symbolised by women and men typically not wanting to use condoms: ‘I will make an example with the guys I hang out with, they say they don't use condoms with their main partners because they trust them, then the other girlfriends they don't care about, they use condoms with them’ (Participant, focus group 1). Introducing condoms into these relationships signalled a breakdown in trust and love, tantamount to admitting these relationships were not the monogamous idealised relationships men sought to portray and sustain.

#### Violence and control over female partners

In the FGDs and interviews, men spoke openly about how they used a range of techniques to control their female partners, including violence. Men's use of violence against their partners was closely linked to a range of controlling behaviours and almost always positioned as an active strategy by men to achieve respect and social position that they felt they had been denied.

Men's controlling behaviours towards their main partners attempted to limit women's autonomy. Often this was done, according to men, because they feared women would ‘cheat’ on them with other men; an inability of men to control their partners, devalued men's sense of themselves. Controlling behaviours included checking cell phone messages, screening calls, and making calls throughout the day and night and expecting immediate answers. Sandile explained he trusted his main girlfriend because no matter what time he called she would answer her phone and talk to him:

Sandile: Since my girlfriend stays very far from my community, so like every time I call her she will always pick up my calls, and we talk for a very long time. It does not matter what time I call, she does not have a problem, like making excuses if she has a man around her you know and all that. I have never caught her doing anything wrong, like with a man [cheating], and all the silly things. (IDI, 24, temporary jobs)

When men's controlling behaviours failed to achieve what was wanted, young men readily described using violence as a way of re-establishing both the gender order – women's subordination to men – as well as re-establishing men's respectability within a social hierarchy, as Sandile emphasised when asked why men were violent to their partners:

Sandile: I may not explain exactly why but, from what I have observed, it is because of the girlfriends that misbehave, then that leads to them getting a beating, like a man would say: ‘You are misbehaving, you don't respect me’. (IDI, 24, temporary jobs)

Participants identified a wide-range of ways in which they felt women disrespected them and where violence could legitimately be used to reassert men's respect and dignity. Many focused around men's concerns that women would cheat on them. Other ‘reasons’ included women's growing economic autonomy and a concern that this would lead to women disrespecting men, with violence used to reassert male power:

Mthobisi: When a woman, like she is working, and I am not working, and she starts disrespecting and being rude to me, we then fight then like I end up hitting her because I try to defend my dignity as a man. (IDI, 22, rents a room, sister supports)

Women refusing to have sex with a male partner also was potentially a source of violence, reflecting ideas of sexual entitlement, although many men said this was something they accepted. One participant, Gwedi, described how one evening his second female partner (not his main partner) came over, but did not want to have sex with him. Gwedi felt that the only way of dealing with this affront (which also implied that she had another partner) was to beat her as he had been humiliated:

Gwedi: I had to lay a hand on her [hit her] because of what she did. She came to my house at night drunk, and I wanted to have sex with her, and she denied me sex because she was drunk … then I waited until the morning, and at that point it had been days since I had had sex with her, so like now in the morning like I wanted some, because I had been longing to have sex with her, so she pretended she was going outside to pee [there are only outside toilets] … I realised she was not coming back, she was going home. So I chased after her I then grabbed her, I slapped her for the fact that she was running away, but I ended up not sleeping with her, because she was then talking about police and all that [laughing]. So I beat her up for making me a fool, because she should have said she does not want to have sex with me straight up, you see what I mean? (IDI, 24, petty drug seller)

Violence and controlling behaviours enacted by men against their female partners were widely described by men as an attempt to reassert their dignity and respect in relation to women.

#### Multiple sexual partners

The central role for young men living in urban informal settlements in seeking multiple sexual partners to establish their identity was evident. Having multiple partners was normalised. For Thokozani, it was something that men just needed:

Thokozani: But you know a woman can have one or two partners. But men cannot live without having more than one partner and there are very few of them that can live with only one. (IDI, 19, supported by parents)

While a few participants suggested a ‘cultural’ basis for multiple sexual partnerships, the major emphasis was that multiple sexual partners were a way of earning respect from their peer-group. One focus group participant commented on why men had many partners: ‘they can be complimented for being a real man’ (focus group 3). Another, China, similarly suggested that having multiple girlfriends earned you respect, affirmation, and dignity from your peers:

China: If you have one girlfriend you are a coward; most of them do it for pride and they do it so that they can get respect and for the dignity and when you have many girlfriends it means you get respect. (Focus group 3)

The performative nature of seeking and securing multiple sexual partners was particularly evident in the way short-term, one-off sexual encounters were described by these men. Mthobisi described how these were linked to parties and drinking alcohol and proving to your friends that you were able to be successful sexually:

Mthobisi: You know at the parties, condoms are the last thing people think of when they are drunk and then you go and have sex with the girls and end up contracting HIV because of the fact that you were trying to please friends. (IDI, 22, rents a room, sister supports)

Having multiple sexual partners was a public performance of heterosexuality, proof of desirability, and thus masculinity. As such, they provided a pathway open to these young men for building up a sense of respect.

#### Defence of honour: men's violence to other men

A final way men talked about achieving public respect and proving masculinity was demonstrating a readiness to defend their honour through violence towards other men. Typically alcohol was also involved; however, violence occurred when men felt they had been slighted by another man and needed to defend their dignity. Mthobisi described how fighting emerged because men felt the need to not lose face or back down if they had been disrespected:

Interviewer: Who do men get violent towards?

Mthobisi: Towards other men, if like you have lowered his dignity as a man…

Interviewer: Can you give me an example?

Mthobisi: If you come and look down upon me and be rude, swear or talk nuisance to me, obviously I will have to defend my dignity I will then stand up and confront you and if we fight, we fight.

Interviewer: Why do men get violent?

Mthobisi: Most of the time it's because they are drunk or it is because they are just short tempered, there are those that are like that who when you speak to them they just answer you for the sake of just answering you, they are not open.

Interviewer: Why do they fight with each other?

Mthobisi: It's pride my brother you know men value their dignity, I will also return the favour hurt and injure you just so I can get my dignity back as a man. (IDI, 22, rents a room, sister supports)

Similarly Goodman described how an argument could easily escalate into a fight, particularly if alcohol was involved:

Interviewer: So who are men violent towards?

Goodman: Each other.

Interviewer: Why?

Goodman: You know you will find that one person steps on the other and the one being stepped on would say ‘can't you see you stood on my toe’ and the second guy would say: ‘I'm sorry’, then the first one would try and provoke the other one since he is drunk and because maybe he has a grudge with the second guy or something like that. And maybe the second one would end up saying: ‘I said I'm sorry, what do you want me to say’ and if the first one keeps pushing, then the second one would say, ‘what are you going to do’ then the fight starts over that little incident. (IDI, 25, supported by mother)

Men's violence to one another was very public and linked closely to men's overarching concern to position themselves within a dominant gendered hierarchy. While alcohol often fuelled this violence, men felt they could not ‘back down’ without losing respect.

## Discussion

In this study, we have sought to understand how young men in two urban informal settlements in eThekweni, South Africa, construct and maintain one particular set of social and sexual identities in the face of high levels of unemployment and poverty, recognising the relational nature of masculinities and their multiplicity in any given setting. Broadly we have suggested that while these young men aspired to a ‘traditional’ worker masculinity forged in the 1970s industrialisation in South Africa, with its emphasis on economic power to setup and sustain a household, including assertion of power over women and children (4), their ability to do so was severely compromised because they lacked the material power to do so.

In turn, young men sought out other ways of building their sense of power and respect in response to the life challenges they faced and their inability to obtain other sources of respect. Principally young men ‘on the wrong side of history’ (42) established a subordinated masculinity, much the same as outlined by Wood and Jewkes (34) in the Eastern Cape of South Africa. This youth masculinity prioritised, in lieu of power through setting up and sustaining a home, education, or wealth, power in spaces that young men could achieve, most evidently through asserting power and control over women, particularly main sexual partners and seeking multiple sexual partnerships and violence towards other men. These practices, similarity to those described by Wood and Jewkes (34), suggest a commonality of how marginalised youth in South Africa attempt to position themselves within a gender hierarchy in contexts of poverty and unemployment.

That these sources of power are the only ones available to young men in these contexts, emerges from a long history of economic, political, and social exclusion of, and violence directed towards, young Black South Africans (43), and the continued dominance of conservative patriarchies in South Africa, as well as the inter-generational production of trauma and violence, experienced by many young men (31).

Of note, however, is that young men expressed significant emotional investment in their long-term relationships with main female sexual partners. This contrasts sharply to much writing on young men, which emphasises the extractive nature and lack of emotional engagement in men's relationships (8, 28). It also points to how men draw on a range of discourses of masculinity in different relationships (19) suggesting that there may be discourses and opportunities for change already embedded in men's everyday practices. However, as Wood and Jewkes (34) suggest, men's investment in these relationships is also a way of demonstrating masculinity, with men's ability to retain and control women being critical to them.

The youth masculinity described in the data also contrasts with that described by Hunter (4, 35). Hunter suggests that in the face of HIV, young men in KwaZulu-Natal are starting to modify their sexual behaviours as organic responses to risk. However, our data suggest that for many men, this is not happening, with the pressure to achieve respect and social positioning in the gender order outweighing other priorities.

For young men in urban informal settlements, their sense of masculinity and positioning within the gender hierarchy was very publicly achieved and evaluated; something men ‘wore on their sleeve’ and performed. In very different contexts Vandello and Bosson (44) suggest that masculinity is, in the most part, extremely precarious, something that is ‘Hard Won and Easily Lost’. While not directly emphasising masculinities performative nature, such an argument resonates with Butler's (45) notion of gender being a performative category (albeit one performed within material and political constraints). Indeed, the young men in this study certainly continued to perform their masculinity on a daily basis, recognising how they were publicly evaluated. This may have been compounded by the very public nature of everyday life in the two informal settlements. As young men lived in small, one room shacks, often shared with others, they had few private spaces into which they could retreat and enact alternative forms of masculinity, outside the gaze of dominant social and gender norms. While much writing has explored how place shapes health (6, 11, 12, 29), little has considered how the public nature of life in urban informal settlements and the lack of private spaces may contribute to certain configurations of gender practices emerging.

The argument set out in this paper has three implications for working to reduce violence and HIV risk more broadly with men. First, even within the youth masculinity that we describe, there existed a number of contradictions and opportunities to support more gender equitable – or at least less harmful – masculinities, ones emphasising trust, love, and long-term commitment. As has been pointed out (19), these provide discourses for interventions to draw on and build from and point to the fluid and multiple nature of masculinities in any given setting.

Second, given the way a youth masculinity coalesces around a number of particular practices, interventions need to work around multiple issues if they are seeking to reduce violence and HIV risk. It is unlikely that changing men's violent behaviour will occur outside of working with them around alcohol use, drug use, multiple sexual partnerships, because these all coalesce around a particular form of youth masculinity.

Finally, as other studies have suggested, violence and other HIV-related risk practices partially emerge from young men's exclusion from the global capitalist economy 46. With a dominant approach to achieving respect cut off for these young men, they cast around for alternative pathways; one of which included what we describe as a youth masculinity. Work from the global peripheries of the capitalist system, including Mozambique (26) and Brazil (18), all point towards how men's violence is implicated in these processes of exclusion (often overlapping with racism). Yet similar dynamics are also seen within the heart of global capitalism. As Bourgois (27) outlines in his ethnography of drug dealers in New York, young Porto Rican men excluded from the capitalist economy secure respect through the only available pathways, dealing drugs and public and private uses of violence. Given these global processes are inflected with local dynamics, there remains much to be learnt about what building young men's livelihoods would look like and whether this would have any bearing on violence and HIV-risk behaviours. More work is also required on how best to work with young men, invested in a contemporary form of youth masculinity which prioritises violence, control, and multiple sexual partnerships to support these young men to change and develop less harmful forms of masculinity in the contexts of poverty and significant life challenges.

## Footnotes

1 Information provided: IDI (in-depth interview), age, primary source of income.
